# Estrogen biosynthesis in cultured skeletal muscle cells (L6) induced by amino acids

**DOI:** 10.1186/s12263-019-0652-8

**Published:** 2019-11-12

**Authors:** Britt-Marie Iresjö, Andreas Landin, Claes Ohlsson, Kent Lundholm

**Affiliations:** 1 Surgical Metabolic Research Lab, Department of Surgery, Institute of clinical sciences, Sahlgrenska Academy, University of Gothenburg, Gothenburg, Sweden; 2 Department of Surgery, Sahlgrenska University Hospital, Region Västra Götaland, Gothenburg, Sweden; 3 Department of Internal Medicine and Clinical Nutrition, Institute of Medicine, Sahlgrenska Academy, University of Gothenburg, Gothenburg, Sweden; 4 Department of Drug Treatment, Sahlgrenska University Hospital, Region Västra Götaland, Gothenburg, Sweden

**Keywords:** Estrogenic steroids, Steroid biosynthesis, Skeletal muscle cells, Amino acids, BCAA, MVD enzyme

## Abstract

**Background:**

Previous investigations have indicated upregulation of gene expression in cellular pathways related to the biosynthesis of steroids in response to amino acids (AA) in skeletal muscle cells. This suggests AA as modulators of de novo synthesis of sex steroids for muscle growth and improved functional capacity. The aim of the present study was to investigate if increased availability of amino acids induced biosynthesis of sex steroids in skeletal muscles.

**Methods:**

Confluent L6 muscle cells were cultured in media with various AA concentrations (0.3 or 9 mM AA or 2.1 mM branched-chain (BCAA) only), following pre-culture in serum-free medium. Sex steroids were quantified by gas chromatography-tandem mass spectrometry (GC-MS/MS). Mevalonate (diphospho-) decarboxylase enzyme (MVD) was quantified by Western blot.

**Results:**

The experiments confirmed that estradiol and estrone increased in both L6 cell lysates and in conditioned media at the end of experiments on confluent cells, while progesterone or androgenic steroids were not detected in either cell lysates or culture media. Estradiol (+ 31 ± 3%) and estrone (+ 18 ± 4%) increased significantly in cells cultured at 9 mM AA (*p* < 0.001 vs. 0.3 mM AA, *n* = 10). Similarly, MVD protein increased at 9 mM AA (*p* < 0.001 vs. 0.3 mM AA, *n* = 17). An addition of BCAA alone to media increased MVD-protein levels to the same extent as all AA (*p* < 0.01 vs. 0.3 mM AA, *n* = 3).

**Conclusion:**

Female sex steroids and MVD enzyme production increased significantly in response to amino acid availability. The results indicate a role of amino acids as modulators of local muscle estrogen synthesis in muscle cells from rats at feeding.

## Introduction

It is well known that skeletal muscles are responsive to steroid hormones such as androgens and estrogens to promote muscle protein synthesis and hypertrophy [1, 2]. Anabolic effects by androgens are well known [3], while effects by estrogens on skeletal muscle anabolism were discovered more recently [4]. Generally, steroid hormones are produced in the adrenals and gonads for circulation to various tissues to promote endocrine effects. However, it is also well known that several tissues, including skeletal muscles, express enzymes capable of local tissue synthesis of sex steroid hormones [5, 6]. The capacity of muscle cells to convert inactive hormone precursors, present at high blood concentrations, into active hormones has also been demonstrated in vitro and in vivo [6], particularly related to the local activation of steroidogenesis following both acute and long-term exercise programs [7–9].

In addition to the abovementioned conditions, we observed that amino acid refeeding induced major upregulation of gene expression in cellular pathways related to biosynthesis and metabolism of steroids in cultured rodent L6 muscle cells [10]. The upregulation of enzymes in the mevalonate pathway for the production of cholesterol, in combination with the upregulation of several hydroxysteroid-dehydrogenase enzymes, for conversion to active forms of sex steroids suggests that amino acids may control intracellular biosynthesis of sex steroids in skeletal muscles [10]. It is however yet unknown to what extent the availability of amino acids may increase intracellular biosynthesis of androgenic and estrogenic steroids in skeletal muscles. The purpose of the present study was therefore to evaluate to what extent the provision of extracellular amino acids may increase muscle intracellular production of sex steroids.

## Methods

### Cell cultures

**Fig. 1:**
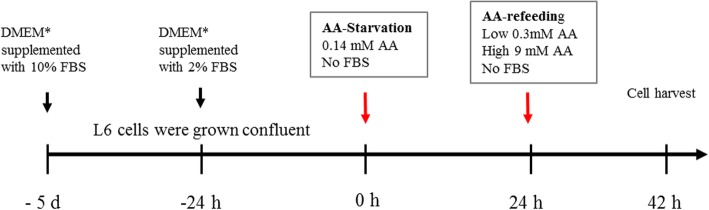
Timeline of the cell culture process. L6 cells were cultured in AA starvation media with low amino acid concentrations (0.14 mM) at the start of experiments. Cell culture media were replaced after 24 h, and cells were incubated in “refeeding media” for 18 h. AA refeeding medium is comparable to DMEM except for modified amino acid concentrations. An asterisk denotes DMEM = Dulbecco’s modified Eagle’s medium, high glucose (contains 9 mM AA and 4.5 g/L glucose). FBS = fetal bovine serum

All experiments were performed on the established rat L6 skeletal muscle cell line (ATCC CRL-1458) using an amino acid starvation-refeeding model as described in detail elsewhere (Fig. 1) [10, 11]. Briefly, L6 skeletal muscle cells were grown confluent in standard cell culture media (Dulbecco’s modified Eagle’s medium with 4.5% glucose (DMEM), supplemented with 10% fetal bovine serum (FBS), 100 IU/ml penicillin, 100 μg/ml streptomycin, and 2 mM glutamine (4–5 days). Culture media were then changed to DMEM supplemented with 2% FBS and cultured additionally 24 h. At the start of experiments, cells were rinsed and media were changed to “starvation medium” with very low amino acid concentrations (0.14 mM) and without FBS or antibiotics. Media were replaced after 24 h, and cells were then incubated in “refeeding media” for 18 h. Refeeding media contained either low amino acid concentrations (0.28 mM, low AA), high amino acids (9 mM, high AA), or branched-chain amino acids (BCAA, 2.8 mM), without FBS or antibiotics. Nine millimolar AA corresponds to concentrations in standard DMEM, equal to approximately twice the plasma levels in humans following meal-feeding [12]. BCAA medium contained increased concentrations of BCAA, while the remaining amino acids were provided at 0.14 mM. Appropriate amino acid levels in media on starvation-refeeding experiments have been confirmed in earlier work to provide cell conditions with low protein turnover (low AA medium) and significantly increased protein translation (high AA medium) without significant cell proliferation confirmed by microscopy [11]. In statin experiments, simvastatin (S-6196, Sigma Aldrich, Saint Louis, USA) or mevinolin (M-2147, Sigma Aldrich, Saint Louis, USA) were added to the final medium at concentrations of 5 μM and 10 μM [13, 14]. L6 skeletal muscle myoblasts were seeded in six-well plates (immunoblotting) or 10-cm petri dishes for steroid quantification by mass spectrometry. Experiments were performed on cell passages 5–25. All products for cell culture were supplied from Sigma Aldrich, Saint Louis, USA.

### GC-MS/MS quantification of sex steroids

Sex steroids (estradiol, estrone, testosterone, dihydrotestosterone (DHT), progesterone, androstenedione, and dehydroepiandrosterone (DHEA)) were quantified by high sensitivity gas chromatography-tandem mass spectrometry (GC-MS/MS) as described in detail elsewhere [15]. Aliquots of either pre-culture or conditioned cell culture media (450 μl, *n* = 4/group) were mixed with internal standard (50 μl) and 0.5 M ammonium acetate (500 μl). Steroids were then extracted by 1-chlorobutane as described [15]. Aliquots of cell lysates (450 μl, *n* = 10/group) were similarly processed. Cell lysates were obtained by scraping cells from two cell cultures (approximately 2 × 10^7^ cells) in 350 μl RIPA buffer (50 mM Tris pH 7.4, 150 mM NaCL, 0.1% SDS, 1% Igepal™ CA-630, 0.5% deoxycholic acid). Cell lysates were weighed, left on ice for 30 min, and mixed and pipetted in 450 μl aliquots for quantification. Results are expressed as pg steroids/10 ml of media or as pg from each cell extract. Results are also presented as the relative increase in percent. Experiments were repeated four times with two to three independent samples each time. Lower limit of quantification of estrone was 0.5 pg/ml, estradiol 2.5 pg/ml, progesterone 74 pg/ml, androstenedione 12 pg/ml, DHT 2.5 pg/ml, and testosterone 8 pg/ml.

### Cell numbers

Cell numbers in culture experiments were estimated in separate parallel cultures by crystal violet staining of cell nuclei. Cells were seeded in 48-well dishes, grown confluent, and thereafter starved-refed as described (Fig. 1). At the end of experiments, the medium was aspirated and cells were fixed in glacial acetic acid: 99.5% ethanol (1:3) for 15 min, thereafter air dried, and then stained in 0.2% crystal violet in 20% methanol for 10 min, followed by de-staining in 20% methanol and air-dried. The stain was dissolved in 1% sodium dodecyl sulfate. Aliquots were measured in a 96-well plate at 570 nm [16].

### Immunoblotting of mevalonate (diphospho-) decarboxylase enzyme (MVD)

Cells were lysed by scraping cells in ice-cold RIPA buffer with an addition of complete protease inhibitor cocktail (Roche Diagnostics GmBh, Germany). Lysates were transferred to test tubes, left on ice for 30 min, and then centrifuged 15 min at 10000×g at + 4 °C. Supernatants were collected and protein concentration was determined by the Bradford method using albumin as the standard (Quick Start Bradford Protein Assay, Bio-Rad Laboratories Inc.). Thirty micrograms of protein from each supernatant were separated in 4–12% NuPage Bis-Tris minigels using MOPS buffer system, according to the manufacturer’s instructions (Life Technologies), and transferred to 0.2 μM PVDF membranes. Membranes were blocked in 10% non-fat dry milk in Tris-buffered saline containing 0.05% Tween-20 for 2 h (TBST). Membranes were then incubated in primary antibody over night at + 4 °C (Anti-MVD (H-11), Santa Cruz Biotechnology Inc. sc-376975) followed by TBST washes and incubation with secondary peroxidase-labeled anti-mouse ab. for 60 min at room temperature (Na931vs, GE Healthcare). Both primary and secondary antibodies were diluted in 3% non-fat dry milk in TBST. Blots were developed using ECL Prime Western Blotting Kit according to the manufacturer’s description (Amersham Biosciences, UK). Chemiluminescent emission signals were captured using ChemiDoc XRS imaging system (BioRad Laboratories, Sundbyberg, Sweden) and quantified (Quantity One software v 4.6.4, Bio-Rad Laboratories AB, Sundbyberg, Sweden). A control sample was loaded at two lanes on each gel for normalization of signal intensity across blots. Optical density is expressed as arbitrary units relative to the control sample. After detection of chemiluminescent signals, gels were stained in Ponceau S to ensure equal protein loading of samples. MVD protein appeared as a single band at approximately 43 kDA (Fig. 3), while hydroxysteroid dehydrogenases (HSD) appeared in multiple bands (Anti-HSD17B1, PA5-42058, Thermofisher Scientific (results not shown)).

### Microarray experiments

The identification and quantification of upregulated RNA transcripts above fold change 2.0 have been reported in details elsewhere [10]. Additional results from such experiments are now reported in the “Results” section regarding significantly upregulated transcripts above fold change 1.5, related to sex steroid pathways.

### Statistics

Results are presented as mean ± SE. Statistical analyses among two or several groups were performed by ANOVA, with post hoc comparisons using Fisher PLSD test in multi-group comparisons; *p* < 0.05 was considered statistically significant in two-tailed tests.

## Results

### Concentrations of sex steroids in L6 cells and cell culture media

**Fig. 2:**
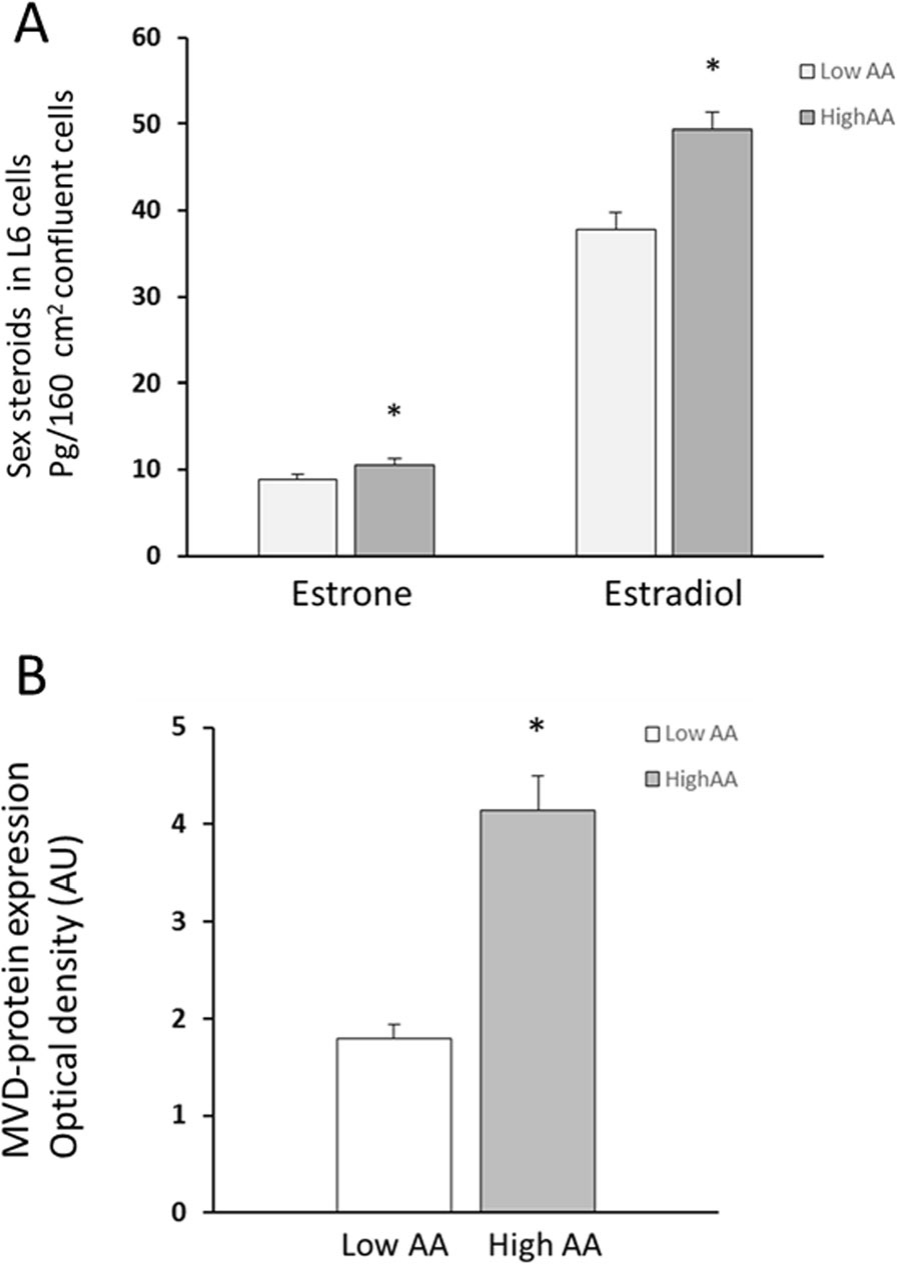
**a**, **b** Effect of amino acids on estrogenic steroids and MVD protein levels. Quantity of estrogenic steroids (**a**) and MVD-protein levels (**b**) increased in L6 cells when cultured in the presence of high AA concentrations (9 mM) compared to low AA concentrations (0.3 mM) in the culture media. Estrone + 18 ± 3%, estradiol + 31 ± 4%, *n* = 10/group in **a**, and *n* = 17/group in **b** * *p* < 0.001 vs low AA. Steroid concentrations were determined by GC-MS/MS and MVD proteins by Western blot as described in “Methods” section. Cell numbers following low and high amino acid exposures were equal as confirmed by crystal violet staining (≈ 2 × 10^7^ cells in **a**)

Estradiol and estrone were present in L6 cell lysates as well as conditioned media, while testosterone, DHT, progesterone, androstenedione, and dehydroepiandrosterone were below detection limits in both cell lysates and conditioned culture media. Estradiol amounts were approximately five times higher than the estrone levels in L6 cell lysates. Cell content of estradiol increased significantly in cells cultured in the presence of high AA compared to low AA (49.4 ± 2.0 pg vs. 37.8 ± 2.0 pg, *p* < 0.001, *n* = 10), while estrone levels increased borderline (10.5 ± 0.7 pg vs. 8.9 ± 0.5 pg, *n* = 10, *p* < 0.08) (Fig. 2a). The relative increase of both estradiol (+ 31 ± 3%) and estrone (+ 18 ± 4%) in paired samples was highly significant in cell lysates (*p* < 0.001).

Pre-culture media did not contain any detectable steroids (*n* = 4). Estradiol and estrone were present in cell culture media collected at the end of experiments (18 h exposure to cells). Steroid concentrations in media were present at approximately the same ratio as found in cell lysates, with estradiol concentrations approximately five times higher than estrone concentrations (estrone 19 ± 2 and 28 ± 6 pg/10 ml media; estradiol 92 ± 8 and 136 ± 14 pg/10 ml media, from low and high AA treated cells, respectively, *n* = 2/treatment). Cell numbers following low and high amino acid exposures were equal as indicated by crystal violet staining in parallel experiments (*n* = 8/group, low AA 100 ± 3%, high AA 101 ± 2%).

### Effect of amino acids and statins on mevalonate (diphospho-) decarboxylase enzyme (MVD)

**Fig. 3:**
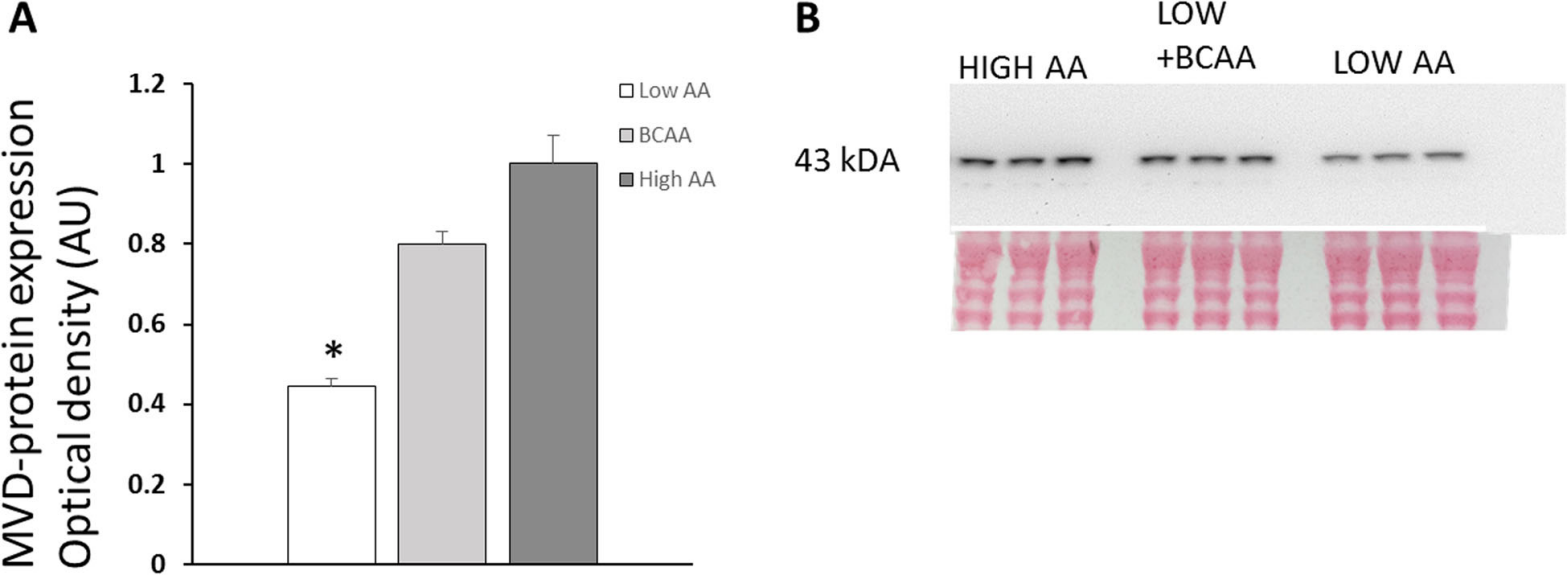
**a**, **b** Western blot of MVD proteins. **a** MVD-protein levels increased almost equally when confluent L6 cells were cultured in the presence of BCAA concentrations only (2.8 mM), compared to all amino acids at high (9 mM) and low amino acid concentrations (0.3 mM). * *p* < 0.01 vs BCAA and high AA; *n* = 3 per group. **b** MVD proteins appeared at expected 43 kDa as a single band. Equal amounts of total protein/sample were applied (30 μg) and confirmed by Ponceau S staining of membranes. Chemiluminescent signals were quantified in ChemiDoc imaging system and Quantity One software

MVD-protein levels increased significantly in L6 skeletal muscle cells cultured in high AA medium compared to low AA concentration (+ 131%, *p* < 0.001, *n* = 17/group; Fig. 2b). MVD-protein levels also increased significantly compared to low AA when culture media contained elevated BCAA concentrations only (Fig. 3, *p* < 0.01, *n* = 3/group). The treatment of L6 cells with statins (mevinolin 5 and 10 μM, or simvastatin 5 and 10 μM, *n* = 6–9/condition) did not change MVD-protein levels in the presence of either low or high medium AA concentrations (*p* > 0.05, results not shown). This suggests that the control of MVD enzyme production in skeletal muscle cells during present experimental conditions was related to increased amino acid availability rather than feedback control from decreased cholesterol levels as shown in other cell types, where statin inhibition of cholesterol production was reported to increase mevalonate pathway enzymes levels, including MVD [17, 18].

### Microarray results

Microarray analysis was performed on RNA from eight samples: four low AA- and four high AA-treated L6 skeletal muscle cell cultures. Significantly altered transcripts in GO (gene ontology) categories “steroid biosynthetic process” and “steroid metabolic process” were reported with a fold change > 2, transcripts that mainly belonged to mevalonate pathway enzymes for cholesterol synthesis [10]. Presently, we report transcripts with relevance for sex steroid biosynthesis, cholesterol intracellular transport, and cholesterol transfer to the mitochondria that showed a magnitude of alteration > 1.5 fold change (*p* < 0.05). These transcripts are: DBI, NM_031853 up 1.88; Transcripts of StAR-related lipid transfer domain containing genes (Stard3, NM_001014229 down 1.9; Stard 4, NM_001106159 up 3.1; Stard6, NM_001007627 up 1.6; Stard10, NM_001013069 down 2.9). Transcripts of Hydroxisteroid- dehydrogenases (Hsd17b1 NM_017080 up 1.9; Hsd17b7, MN_017235 up 3.1; Hsd17b12 NM_032066 up 1.8).

## Discussion

The present study was designed to evaluate intracellular biosynthesis of steroids induced by increased extracellular amino acid availability in muscle cells, to support our previous observations based on gene transcription reflecting enzyme productions for steroid synthesis [10]. Thus, our previous microarray experiments showed significant enrichment of gene-transcripts in gene tandem categories related to steroid biosynthesis and metabolism [10]. Transcripts of all enzymes in the mevalonate pathway for the production of cholesterol were significantly upregulated in the presence of high extracellular amino acid concentrations (fold changes from 2 to 6) [10], while transcripts of enzymes involved in the final conversion to biologically active forms of sex steroids showed overall lower magnitudes, although yet significant upregulations (1.5–3 fold; the “Results” section). Similar differences in transcript levels were also displayed by genes related to intracellular cholesterol trafficking or transfer of cholesterol to inner mitochondrial membranes, where StarD6 is reported to interact with the mitochondrial membrane moving cholesterol from outer to inner mitochondrial membranes, necessary for initiation of steroid synthesis [19, 20]. It was thus appealing to suggest that amino acids may induce de novo steroid biosynthesis in muscle cells.

Our present experiments demonstrate the synthesis of estrogenic steroids by amino acids, without similar effects on androgens, which agree with our microarray data, where 17β-hydroxysteroid dehydrogenase 1, 7, and 12 were increased while HSD3 transcripts were not altered in the presence of amino acids in L6. A lack of androgenic steroid production is unclear but may be explained by a lack or low levels of the classical intracellular androgen receptor in L6 cells [21]. However, despite this, L6 cells were reported to respond to testosterone, effects proposed to be mediated through non-genomic pathways via G-protein coupled receptors [21].

The capacity of muscle cells to convert inactive hormone precursors in blood circulation into active steroids is well established [6], with relevance for local steroidogenesis following both acute and long-term exercise [7, 8]. Observations of increased testosterone production, as well as the reversal of the age-associated decline in muscle sex steroid hormone levels, have been reported following resistance training in men [7]. However, that steroids may be synthesized de novo in the presence of amino acids in muscle cells has not been reported to our knowledge.

Cholesterol, the sole precursor of all steroid hormones, may be synthesized within most cell types in a series of enzymatic reactions known as the mevalonate pathway. Initial substrates in this pathway are acetyl-CoA and β-hydroxy-β-methylglutaryl-CoA (HMB-CoA); molecules that are produced during the metabolism of ketogenic amino acids, such as the branched-chain amino acids (BCAA), although HMB-CoA is only formed by leucine metabolism. Leucine has been reported to contribute significantly to cholesterol production in the muscle tissue, although oxidation and protein synthesis are considered major metabolic pathways for BCAA utilization in muscle tissue [22]. Moreover, leucine conversion into cholesterol is increased by insulin in skeletal muscles [22]. Such observations agree with our present concept that increased amino acid availability should contribute to local biosynthesis of steroids in anabolic conditions and that BCAA induced metabolism is of major importance as indicated by our earlier experiments on incubated human muscle fibers [23].

L6 is a rat myoblast cell line often used in experimental studies of muscle protein synthesis in proliferation and differentiation of muscle cells [21, 24, 25]. Our present and previous studies were performed in a cell culture model of confluent L6 skeletal muscle cells exposed to an initial period of amino acid starvation followed by amino acid refeeding at low and high concentrations, confirmed to initiate muscle protein synthesis without the start of significant cell proliferation [10, 11]. The branched-chain amino acids are regarded as important for the activation of muscle protein synthesis. Also, the catabolism of BCAAs is promoted during exercise, which is associated with increased local steroidogenesis in muscles [7, 8, 26]. We therefore tested if the increased provision of branched-chain amino acids alone could alter enzyme levels related to cholesterol and steroid biosynthesis. Accordingly, we found that MVD protein increased significantly in L6 cells when only branched-chain amino acids were provided at increased concentrations in the culture medium. MVD is a limiting enzyme in the mevalonate pathway for the production of cholesterol and was among most upregulated transcripts in our genomic experiments [10]. We tried to quantify 17β-HSD1 proteins responsible for the conversion of estrone to estradiol, but results were not clear cut conclusive with several protein bands appearing at multiple molecular weights. Similar findings were reported for other enzymes in the 17β-HSD family where multiple and different molecular weights were found depending on tissue sites [27]. Thus, such findings may indicate differently spliced versions of enzymes in various tissues, suggesting additional control sites of intracellular steroid productions.

In summary, our present experiments confirmed increased de novo production of sex steroids in response to the increased availability of amino acids to L6 muscle cells, as indicated by gene transcription experiments [10]. Estrone and estradiol were synthesized by the L6 cells and appeared subsequently at increased concentrations in the cell culture medium following amino acid provision. This suggests that local muscle productions of steroids may be autocrine and perhaps principally different from reported findings in exercise-mediated production of steroids subsequently to systemically increased steroidogenesis [7]. Local muscle production of steroids may thus be important for the stimulation of muscle satellite cell proliferation [28]. Studies to evaluate to what extent amino acids may induce steroid biosynthesis in human muscles in vivo are ongoing in our laboratory.

## Data Availability

All data generated or analyzed during this study are included in this published article. The microarray datasets referenced in the current study are available from the corresponding author on reasonable request.
